# The Earning and Spending Habits of Male Sex Workers in Lima, Peru

**DOI:** 10.1177/2158244017753046

**Published:** 2018-01-24

**Authors:** Paul E. George, Juan Carlos Bazo-Alvarez, Angela M. Bayer

**Affiliations:** 1 University of California, Los Angeles, USA; 2 Texas Children’s Hospital, Houston, USA; 3 Universidad Católica los Ángeles de Chimbote (ULADECH-Católica), Perú; 4 Peruvian Research Institute of Educational and Social Psychology (PSYCOPERU), Lima, Peru; 5 Universidad Peruana Cayetano Heredia, Lima, Peru

**Keywords:** male sex worker (MSW), Lima, Peru, income, spending, economy

## Abstract

Over the past decade, data have identified male sex work as a potentially viable economic decision; despite this, male sex workers (MSWs) continue to be perceived as group with access to few assets and resources. Using data from a pilot skills— building intervention for MSWs in Lima, Peru, an analysis of the economic characteristics of 209 MSWs is presented. The majority reported livable incomes with median earnings of US$250 per month, 83% earning above the urban poverty line. Interestingly, non-sex work was also an important source of income, especially for the high-earning MSWs. Spending data revealed that a large portion of income went to necessities (55%), luxuries (11%), and gifts (11%), with less toward savings (5%) and studies (1%). Such data on MSWs’ earnings and spending, which suggest that a lack of overall income is not the MSW’s main impediment to escaping poverty, could direct future poverty alleviation and health improvement programs in this key population.

## Introduction

Sex work is defined as the exchange of sexual services for money or goods and as a profession that provides rapid pay without formal training (Visano, 1988). Despite an estimated worldwide revenue of greater than US$180 billion (Prostitution: Prices and Statistics of the Global Sex Trade, 2015), sex workers, especially street-based and male sex workers (MSWs), have historically been stereotyped as “poor, untrained hustlers” or “passive, disempowered victims” who enter the profession as a means of earning a quick albeit often very low income (Bimbi, 2007). These perceptions, however, have changed in recent years and the literature has increasingly recognized that sex work can be a viable economic decision and source of income and employment (Minichiello, Scott, & Callander, 2013). As noted in his review of the changing face of MSW research, David Bimbi (2007) states that “researchers have embraced a new paradigm that respects MSWs’ personal motivations for sex work . . . with the view of sex work as a job and, hence, a valid source of income” (p. 8). The classification of these men as “sex workers” rather than “prostitutes” reflects this paradigm change.

Although it has long been recognized that there is a subgroup of high-end, high-earning MSWs, recent data have supported the notion that even street-based sex work can be a rational economic decision, especially in low- and middle-income countries where other means of employment is often restricted (Scott et al., 2005). Limited self-reported earnings data from street-based MSWs in different global contexts is not typically consistent with the stereotype of these men being poor and destitute, instead identifying livable wages. In Vietnam, for example, MSWs reported earning on average US$266 per month (Biello, Colby, Closson, & Mimiaga, 2014), well above both the poverty line in Vietnam of US$20 per month and the Vietnamese gross national per capita income of US$106 per month (Online Communications Agency of the Socialist Republic of Vietnam, 2011). Similarly, a study from Shanghai reported that 79% of MSWs earned more than 1,000 yuan per month (He et al., 2007), putting them in the top 60% of earners in Shanghai (Chen, Hao, & Stephens, 2010), with 35% of MSWs earning more than 3,000 yuan per month (He et al., 2007), which puts them in the top 20% of the overall population (Online Communications Agency of the Socialist Republic of Vietnam, 2011). Data on street-based MSWs’ earnings in other settings (e.g., Kenya, South Africa, India) have demonstrated similar results of men consistently earning well above the poverty line (Geibel et al., 2008; Richter, Chersich, Temmerman, & Luchters, 2013; Shinde et al., 2009).

Despite data suggesting decent, livable wages, street-based MSWs remain a group with few assets and resources. As a recent review on male sex work around the globe in the *Lancet* notes, the underlying reason for MSWs to become trapped in the profession is poverty (Baral et al., 2014). For example, a 2010 Peruvian study of MSWs reported a median monthly income from sex work of US$172 (interquartile range [IQR] = 100-316) in the street-based group (Bayer, Garvich, et al., 2014). Although these incomes were well above the 2010 national urban poverty line in Peru of US$96 per month (Instituto Nacional de Estadistica e Informatica, 2015), the authors reported that Peruvian MSWs are “just getting by” in the moment with little investment in tangible and future assets or opportunity. Similarly, in a study from Argentina, 77% of MSWs said they could “only manage to get by” (Mariño, Minichiello, & Disogra, 2003).

Negative structural factors, such as poverty, poor access to housing, and discriminatory policy, have been well described as negative health predictors (Sumartojo, Doll, Holtgrave, Gayle, & Merson, 2000). For example, prior research has further described how MSWs’ precarious economic standing has trapped them in sex work and led them to engage in high-risk sexual behavior (i.e., condomless anal intercourse [CLAI]). A qualitative study from the same population of Peruvian MSWs revealed that many street-based MSWs desire to exit the profession but are unable to due to economic restraints (Bayer, Garvich, et al., 2014). These same men reported that they knowingly engage in higher risk CLAI for higher pay. This willingness to enter riskier but more profitable sex work services is echoed in studies with male, female, and transgender sex workers from other settings (Choi & Holroyd, 2007; Infante, Sosa-Rubi, & Cuadra, 2009; Katsulis & Durfee, 2012; Niccolai, King, Eritsyan, Safiullina, & Rusakova, 2013).

This cycle, engaging in potentially self-destructive behavior (such as CLAI) with the goal of staying afloat day-to-day at the expense of future well-being, is an example of the well-described “poverty trap” (Hanlon, 2010). Nationwide implementation of poverty alleviation programs in Mexico (Fernald, Gertler, & Neufeld, 2008), Brazil (Soares, Ribas, & Osório, 2010), and Peru (Jones, Vargas, & Villar, 2008), interventions that aim to break the cycle of the classic poverty trap, have demonstrated reductions in poverty with downstream benefits in childhood school attendance, nutrition, and weight gain. Scientific poverty alleviation trials in impoverished settings have also shown evidence of prevention of tuberculosis (Boccia et al., 2011), stress (Haushofer & Shapiro, 2013), mental health disorders (Baird, de Hoop, & Özler, 2013), and HIV (Pettifor, MacPhail, Nguyen, & Rosenberg, 2012), demonstrating important examples of how poverty negatively affects health.

For Peruvian MSWs, healthier behaviors with regard to the HIV/AIDS epidemic are especially noteworthy, as an estimated 25% of MSWs are infected with HIV, compared with 12.4% among men who have sex with men and 0.4% among the general population nationwide (García, Bayer, & Cárcamo, 2014; Ministerio de Salud [MINSA], Programa Conjunto de las Naciones Unidas sobre el VIH/SIDA [ONUSIDA], 2012; Ugarte Ubillúz, & Arce Rodríguez, 2010). HIV/STI (sexually transmitted infection) risk is especially important for MSWs given that the HIV epidemic in Latin America is largely concentrated in the homosexual and transgender communities (García et al., 2014). Furthermore, MSWs, given their large numbers of sexual contacts (both male and female) and inconsistent condom use, are important potential modes of transmission of HIV, both within and outside of the MSW community (George et al., 2016). Thus, HIV/STI prevention efforts among MSWs can be important public health interventions. Of note, a recent study carried out ethnographic mapping of MSW venues and used the capture-recapture methodology to estimate the number of MSWs in metropolitan Lima. This study estimated that there are 542 MSWs, with a 95% confidence interval (CI) of [475, 609] MSWs, in the metropolitan area (Bayer, Diaz, Malima, & Gorbach, 2014). This is an important estimate for Peru, although it includes only the capital and venue-based MSWs; it does not include those who provide services online or using cell phone—based messages, calls, and applications.

With the background of poverty as a well-described negative health predictor, the seemingly contradictory data that MSWs have access to livable incomes yet remain a group with very few tangible assets led to the following research questions: Do street-based MSWs actually earn livable wages? If so, why do they continue to “just get by” and remain a group with few assets and resources? The aims of this study are therefore to (a) characterize the earnings of a cohort of Peruvian street-based MSWs; (b) determine if their income level correlates with CLAI, HIV, and other STIs; and (c) analyze their spending habits to contribute to the understanding of why MSWs remain impoverished if they do indeed earn a sufficient income.

## Method

The current study is part of a pilot randomized controlled trial (RCT) to assess the initial efficacy of a skill-building center intervention to prevent HIV and other STIs among MSWs in Lima. Specifically, all 210 men in the trial were able to access the skill-building center, including a space to shower, cook and prepare food, do laundry, rest, watch television and use computers and the Internet, and access free medical care and condoms, whereas only those in the intervention arm were able to access personal development, job-training and health prevention group workshops, and individual counseling sessions. The current study is an analysis of the economic characteristics of the study participants. Eligibility criteria included (a) born male and self-identified as male (non-transgender), (b) considers himself a MSW, (c) resides in Lima, (d) age 15 or older, and (e) mentally competent to provide informed consent. All appropriate institutional review boards, specifically the University of California, Los Angeles, and Universidad Peruana Cayetano Heredia (Lima, Peru), reviewed and approved the study. All participants provided their verbal informed consent prior to enrolling and participating in any study activities.

In 2013, an extensive ethnographic mapping process to identify commercial sex venues of MSWs in Lima was carried out (Bayer & Diaz, 2014). This mapping included public plazas and streets, saunas, pornographic video houses, bars, and nightclubs. During 2014, trained, peer-based recruiters revisited these street-based commercial sex venues and invited MSWs to participate in the study. The use of the comprehensive ethnographic map, which maximized the potential sex work venues visited for recruitment, and recruiters familiar with the MSW community, which allowed for recruiting from a large pool of MSWs as opposed to only those who had previously heard of the trial or who took the initiative to come to the center, limited potential selection bias. If initially eligible, the MSW was invited to the skill-building center for additional eligibility screening and the informed consent process. After confirmation of eligibility by the study team and provision of verbal consent by the participant, the MSW initiated his participation in the study.

As part of the study, participants completed baseline behavioral surveys and testing for HIV and other STIs, including syphilis, neisseria gonorrhoeae (urethral, rectal, and oropharyngeal), and chlamydia trachomatis (urethral, rectal, and oropharyngeal). Behavioral surveys were completed using computer-assisted personal interviewing (CAPI), through which trained interviewers asked the participants the questions and completed the participant responses on the computer. The surveys, which took approximately 90 min, assessed several topics, including demographic characteristics, economic measures, and sexual risk behaviors.

Sociodemographic measures were collected in the baseline survey from both the intervention and control arm via self-report. Specifically, the following variables were collected: age and education (chosen because they are variables that influence income), sexual role and orientation (in similar settings, these variables affect clientele and thus potentially income; Bayer, Garvich, et al., 2014), drug and alcohol use (widely known to affect spending habits in many populations; Rosen, Bailey, & Rosenheck, 2003), HIV/STI risk behaviors, living situation, and income data. Alcohol use was collected using the CAGE questionnaire, which is a widely used and well-validated four question screening instrument for alcohol abuse, and coded as positive if two or more questions were answered affirmative (Saitz, Lepore, Sullivan, Amaro, & Samet, 1999). Overall monthly income, which was collected in the baseline survey (data collection: January 2014-August 2014), was measured by asking the men how many jobs (including sex work) they had in the past month and how much they earned from each job. Income from sex work was further characterized by asking the men how much they charge per sexual act for a client. Housing was assessed by asking the men where they currently live, providing them with three options: (a) In a place where I do not pay rent; (b) In a place where I pay monthly rent; and (c) In a place where I pay daily rent to stay. Other information was collected regarding sex work, including age at initiation of sex work, number of clients in the past month (male, female, transgender), number of clients with whom a condom was not used, and sexual role (insertive vs. receptive) during sex work. Each variable analyzed included data from all study participants (no missing data).

Spending data were collected for a subset of participants during the endpoint survey (data collection: March 2015-July 2015). This subset represents 72 of the 209 participants, which included men from the original cohort not lost to follow-up and those who still identified as MSWs. The categories for the spending data were compiled using a focus group of eight MSWs (focus groups took place from January 2015 to March 2015). These MSWs, in conjunction with the study team, constructed 10 spending categories, which were included in the final survey. These questions asked the men where they had spent their money in the previous month, using the following prompts: rent/place to sleep, food, buying clothes and personal hygiene (hygiene products, washing laundry, haircuts), giving money to partner/family/children/friends, partying (discos, alcohol, drugs), transport, communication, studies, trips, savings, and other/don’t remember. If reported spending/savings/giving did not match reported income, men were prompted about the discrepancy and extra money went to the “don’t remember” category. All income and spending data were collected in Peruvian Nuevo Soles but is presented in U.S. dollars (exchange rate for income is 2.8 soles = US$1, March 2014, and for spending is 3.1 soles = US$1, March 2015; XE.com— USD/PEN Chart, n.d.).

Univariate analysis examined the distribution of the men’s demographic, social, sex work, and economic characteristics. These data were also divided via income, with income levels chosen to divide the men into four similar-numbered groups. The above characteristics were further tested for statistical associations with total income. Total income was chosen as the outcome variable (instead of income from sex work) because the goal was to characterize the overall economic situation of the MSWs. Given that a significant proportion of MSWs’ income is generated outside sex work, excluding this revenue from the analysis would have misrepresented the MSWs’ economic characteristics. Generalized linear models (GLM) were used to determine statistical associations between the MSWs’ total income and numerical variables (e.g., age, number of clients) and ANOVA comparison was used for categorical variables (e.g., high school completion, HIV status). Statistical assumptions such as homoscedasticity, linearity, and normality have been assessed for each model or statistical test. An adjustment using *t* test of unequal variance was used in situations without homoscedasticity; the *t* test remained robust given *N* > 30 situations without normality; and although in some cases, nonlinear models were closer approximations than linear models, linear models were preserved due to the close approximations of both models while respecting simplicity and parsimony. Of note, although incomes are presented in Tables 1 and 2 as four categories, for statistical analysis purposes, total income was treated as a single, continuous variable. Finally, a backward stepwise procedure (using log-likelihood ratio tests) was performed to select the best multiple regression model, considering continuous total income as outcome and using each independent variable presented in Table 1 as potential confounders. Robust variances estimated standard errors in the final multiple model. All analyses were performed using Stata 12.1. The *p* values of <.05 were considered significant.

## Results

A total of 209 street-based MSWs were included in the analysis; sociodemographic data can be found in Table 1. The reported median baseline earnings were US$143 (IQR = 71–250) per month from sex work. In addition, MSWs reported significant income from non-sex work jobs, with a median of 23% (IQR = 0–59) of earnings from non-sex work activities. In total, the MSWs reported a median total monthly income of US$250 (IQR = 143–411).

There were differences in all types of earnings (sex work, non-sex work, and total) across the four income groups. Thirty-five men (17% of cohort) reported total earnings of less than the 2014 urban poverty line of US$117 (Instituto Nacional de Estadistica e Informatica, 2015), whereas the top quarter of earners reported more than US$430 per month. About half (122/209, 58%) of the men had complete secondary school, with higher levels of complete secondary education in the highest income (36/50, 72%) versus other income (53%-57%) groups (*p* = .05, *t* test). Overall, only 28% (58/209) of the men had a stable living situation, defined as paying monthly rent as opposed to paying day-to-day or staying with someone for free. Living varied by income group, with 40% (20/50) of the highest income earners reporting stable living, compared with 21% to 25% of the other groups, although this difference was not statistically significant (*p* = .17). The men’s age was not significantly associated with income or living situation (*p* = .61 and *p* = .55, respectively), with the younger cohort (those less than 25 years old) having similar incomes (median income 650 [IQR = 400-1,150] vs. 730 [IQR = 400-1,150]) and stable living, 25% (27/107) versus 30% (31/102), as compared with those men older than 25 years.

Thirty-one men reported additional charges for CLAI (median US$14 extra [IQR = 5-23]). However, charging for CLAI was not associated with an overall higher income (data not presented). Number of sexual partners and number of clients in the previous 3 months were both positively associated with higher incomes in crude analysis. Of note, adjusted analysis performed using the stepwise multiple regression model finally preserved only two variables: being bisexual (*p* = .015, β = –201, 95% CI = [–363, –38.5], GLM) and total number of clients (*p* < .001, β = 6.8, 95% CI = [3.8, 9.7], GLM), both significantly associated with higher total income. Using GLM analysis, the following were significantly associated with total income: income from sex work (*p* < .001, β = 0.95, 95% CI = [0.83, 1.06]), income from non-sex work (*p* < .001, β = 0.93, 95% CI = [0.78, 1.09]), number of sexual partners in past 3 months (*p* < .001, β = 5.2, 95% CI = [2.5, 8.0]), number of clients in the past 3 months (*p* < .001, β = 6.8, 95% CI = [3.8, 9.8]), and amount charged per encounter (*p* < .01, β = 11.6, 95% CI = [2.5, 18.7]).

Eighty-nine men (43%) reported engaging in CLAI in the previous 3 months. STIs were prevalent in this group, with 103 of the 209 MSWs (49%) testing positive for at least one STI, including HIV. HIV status was positively associated with higher total income (*p* = .04, *t* test) and borderline associated with higher income from sex work (*p* = .056, *t* test).

**Figure 1.**
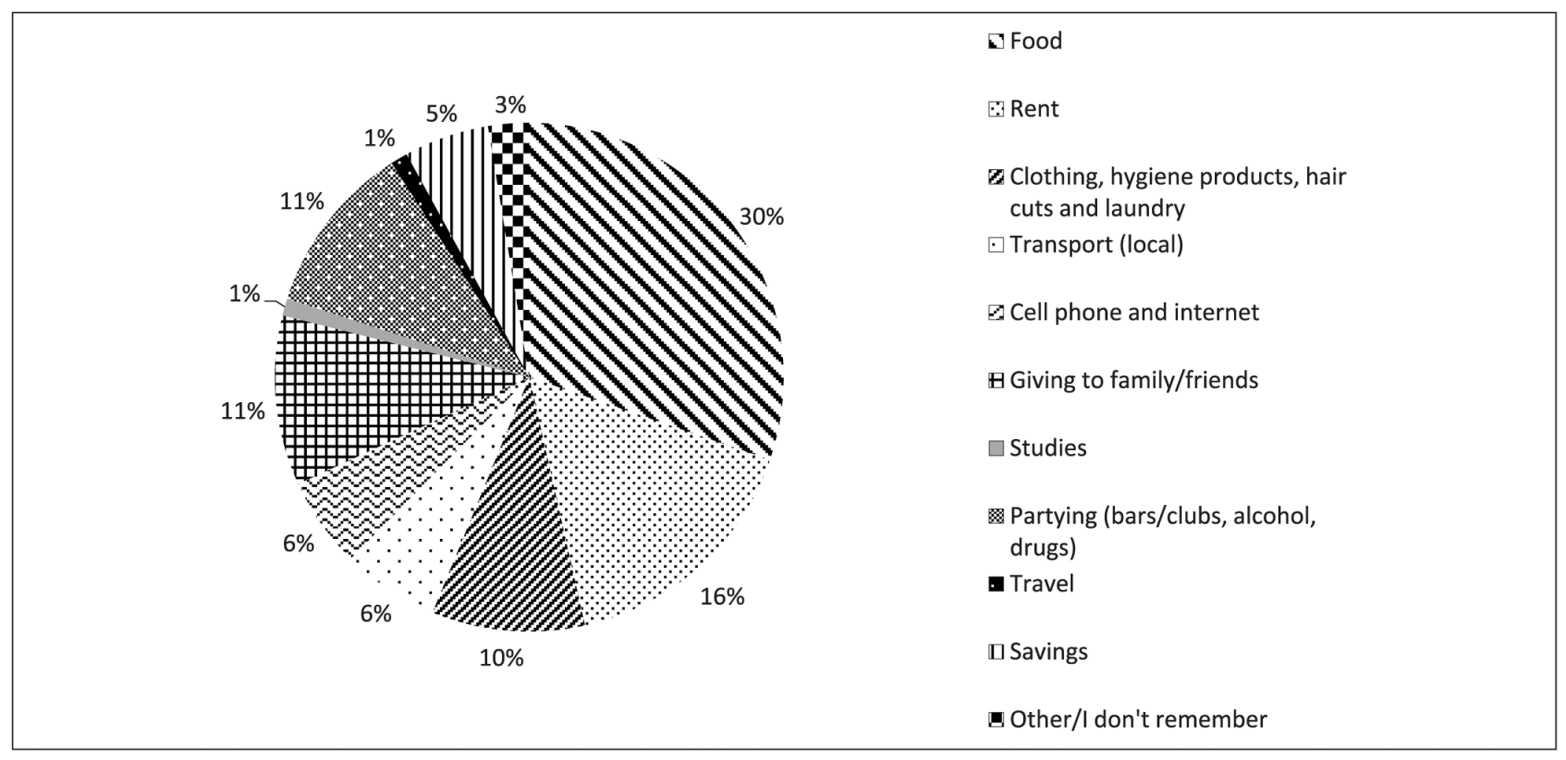
Spending as percentage of total income (M) among a subgroup of male sex workers: Lima, Peru (March 2015).*Note.* The spending and/or saving habits of the cohort taken in aggregate and displayed as percentages. The numerator is the amount of spending per category for the entire group over the previous month, and the denominator is the total amount spent and saved for the entire group over the same time period. For example, the 72 men reported total spending + savings of US$23,500 in the previous month, while spending US$7,000 (30%) on food.

The spending and savings data are presented in Figure 1. Most spending went toward necessities, with food (30%), rent (16%), and personal hygiene, clothing, haircuts, and laundry (10%) accounting for 56% of total spending. Participants also spent significant portions of their earnings on local transport and communications (12%) and giving money to family and friends (11%). Discretionary spending on luxuries, including bars/clubs, alcohol, drugs and travel, was also high at 11%. Savings accounted for 5% of total spending, and investment in studies/job training was minimal at under 1%. Only 3% of spending was unaccounted for. Of note, of all the spending categories, the only category in which there was a statistically significant difference in spending between the younger men (less than 25 years) and older men was partying (mean 14% for the younger men vs. 7% for the older men, *p* < .05, *t* test). The subset of men had similar sociodemographic characteristics (Table 3) as the overall cohort with one notable exception: income. The 72 men reported higher aggregate incomes (median income US$329 [IQR = 184-433]) than the overall group (US$250 [IQR = 143-411]).

## Discussion

This article is the first to our knowledge that provides comprehensive data on the earnings and spending of MSWs, finding that MSWs report livable incomes and significant spending on nonessential items. Despite this, many men are living in poverty, with only 28% reporting stable housing and little money (5% of earnings) going toward future savings. These data, specifically the introduction of more detailed earnings sources and overall spending habits, both of which have been absent from prior literature, are important because poverty reduction has been considered as a potential means of HIV/STI prevention and health promotion in this group. To provide appropriate programs and policy, it is imperative to describe and understand MSWs’ economic situation.

This cohort of Peruvian street-based MSWs reported overall adequate incomes, with a median total income of US$250 per month and 24% (50/209) reporting incomes higher than US$430 per month, both of which are notably above the urban poverty line of US$117 (Instituto Nacional de Estadistica e Informatica, 2015). The findings of this study mirror previous findings, in which many street-based MSWs in other contexts report incomes well above national poverty lines (Biello et al., 2014; Geibel et al., 2008; He et al., 2007; Richter et al., 2013; Shinde et al., 2009). Spending data further revealed that these men were spending under half their income on necessities such as rent, food, and personal hygiene, and a significant portion of men (72%, 151/209) reported unstable living situations.

In Peru and throughout the world, MSWs and especially street-based MSWs bear a disproportionate burden of HIV infection; thus, multifaceted efforts to curb the HIV epidemic among MSWs are warranted (Baral et al., 2014). Poverty and lack of economic opportunity are two identified determinants of HIV risk (Dworkin & Blankenship, 2009) and potential areas of intervention. Living in poverty increases susceptibility to infectious diseases including HIV, reduces access to health care, and restricts options for avoiding high-risk behavior. Mechanistically, poverty is associated with increased rates of malnutrition and parasite infection, which undermine the epithelial barrier and lead to higher rates of infectious diseases, including STIs. The poor are also less likely to have both adequate health literacy and access to health care for STI/HIV, which again increases the community prevalence of such diseases and thus chance of acquiring HIV (Fenton, 2004). Furthermore, poverty can simply be a barrier to procuring HIV/STI medications and even preventive measures such as condoms, again increasing risk. Conversely, improvements in socioeconomic status are associated with improved future outlook and life expectancy and increased preferences for healthier behaviors (Fiszbein, Schady, & Ferreira, 2009). Cash transfers (the direct payment of cash with or without stipulations), one of the most effective means of poverty reduction, have resulted in a reduction of STIs and HIV in other settings, most notably in Africa and with adolescents/young adults (Baird, Garfein, McIntosh, & Özler, 2012; de Walque et al., 2012).

Given these successes, MSWs are considered to be key potential beneficiaries of cash transfers as a means of HIV/STI reduction. In fact, a trial and analysis is currently underway to assess the efficacy of cash transfers as STI reduction tools among MSWs in Mexico (Galárraga et al., 2014). The current results instead suggest that, at least among Peruvian street-based MSWs, a cash influx is not what is lacking. MSWs seem to bring in enough money to avoid poverty. The spending data collected, with 11% of monthly income going to gifts to family and friends and an additional 11% to luxury spending on discos, alcohol, and drug usage, suggest that interventions that teach about proper savings and planning for the future could be of benefit. The finding that older men on average spend less on partying than younger men suggests that interventions directed at younger men could affect saving patterns.

Rather than outside researchers deciding on what intervention seems most logical, however, it is imperative to attain input and buy-in from the community. One of the most successful HIV prevention programs, the Sonagachi project, was a community-based intervention whose approach was driven and implemented by the stakeholders (i.e., sex workers) themselves (Jana, Basu, Rotheram-Borus, & Newman, 2004). Although published behavioral interventions in MSWs are limited, there is reason to be optimistic, as the Sonagachi project as well as a Cochrane review among female sex workers concluded that behavioral interventions can be effective (Wariki et al., 2012). The association between a stable living situation and education presented here adds evidence toward the possible effectiveness of structural behavioral/educational interventions.

Of course, there are other reasons that even with a seemingly sufficient income and proper knowledge and support, MSWs may be unable to lift themselves from poverty. Sex workers have historically experienced stigma and discrimination (Scott, 2003); this fear of discrimination is an important factor that keeps sex workers from using resources that are often free or low cost, such as medical, housing, or other social services that could potentially help in lifting them from poverty (Scorgie et al., 2013). MSWs are also at high risk for both interpersonal (George et al., 2016) and systemic (Decker et al., n.d.; Scorgie et al., 2013) violence, which itself is associated with factors that increase poverty such as physical and mental health deterioration (Beattie et al., 2010), perceived helplessness (Panchanadeswaran et al., 2008), and drug and alcohol abuse (El-Bassel, Witte, Wada, Gilbert, & Wallace, 2001; Niccolai et al., 2013). Furthermore, by virtue of being a sex worker or simply due to being homosexual, MSWs often lack social capital in the form of economic and personal support from family (Niccolai et al.,2013); this informal yet often essential support can be what keeps others off the streets in a moment of economic necessity and is not available to many MSWs (Banerjee & Duflo, 2012). Finally, MSWs may simply be content with their current lives and economic positions and lack motivation to or interest in change.

This study has several limitations that deserve mention. First and most obvious, the income and spending data of the men, given the nature of sex work, are based entirely on recall and without supporting documentation. Social desirability bias could also lead to inaccurate reporting of income and spending. However, analysis of the spending/savings data revealed the men reported monthly spending and savings that correlated very closely with their reported income data, which suggests the men were accurately reporting this data. Social desirability and recall bias could also cause underreporting of sexual risk behaviors, though CAPI was used because previous work with this population proved to be both accurate and the tool preferred by MSWs (Bayer, Garvich, et al., 2014). Furthermore, given the nature of sex work, the men’s income (and consequently spending) likely varies from month to month; in attempting to increase the recall accuracy, we have limited the amount of recall time to 1 month.

The spending data do not represent a random subset of the entire 209-person cohort; instead, these 72 men are those who participated in the spending analysis, which includes those who were not lost to follow-up and who still identified as MSWs during the endpoint survey for the larger RCTin which these men were participating. Although these men had similar sociodemographic characteristics as the entire group, their reported income was higher. As mentioned above, income is variable from month to month and this difference could represent normal variation. Given that the subset of men had higher reported incomes, it is likely they contribute more to savings (thus, the 5% might be an overrepresentation); however, without concrete data on the entire cohort, this is speculation.

Surprisingly, health care spending was very minimal (estimated at US$3 per month or less) and therefore not among these categories. These data do fit within the context of low-resource Peruvians. First, through the Peruvian Ministry of Health, poor and extremely poor Peruvians can receive free care through Comprehensive Health Insurance (SIS). Second, for minor ailments, the men in the current study consistently reported two practices for receiving free or low-cost health care. One, they often received free care from practitioners as part of research studies, a common practice for men who have sex with men as there are several studies with this population in Lima. They also received very low-cost attention by going to a local pharmacy and purchasing low-cost, over-the-counter medicine from a pharmacy technician. The fact that the MSWs themselves did not include health care spending as part of their spending suggests it was a nonsignificant amount; however, it is a limitation not having that data formally collected.

As a cross-sectional study, the findings are not causations but rather correlations. Furthermore, a backward stepwise approach is an exploratory method and the findings may not represent the experiences of all Peruvian MSWs. Finally, caution must be taken when generalizing findings for MSWs in developing countries, especially to non-street-based MSWs who often have different demographic, cultural, and economic circumstances.

## Conclusion

A review from the *Lancet* on HIV prevention concluded that “prevention efforts cannot succeed in the long term without addressing the underlying drivers of HIV risk and vulnerability in different settings” (Gupta, Parkhurst, Ogden, Aggleton, & Mahal, 2008, p. 52). Poverty, as both a known driver of HIV risk and pervasive aspect in the lives of street-based MSWs, is therefore a key element to address in HIV prevention efforts for this population. Our data indicate that the cohort of Peruvian street-based MSWs is not without tangible assets due to a lack of immediate cash flow. Rather, as other studies on similar populations have indicated, it is likely that a combination of personal, structural, and societal factors place this population at a tremendous disadvantage. Thus, interventions that augment cash inflow that have been successful in other settings may not be appropriate for this population; instead, community-based, behavioral interventions that integrate components such as financial literacy and savings promotion could have a more direct and lasting impact on these men’s economic status, which in turn could decrease their risk for HIV and other STI acquisition and improve overall health and well-being.

## Figures and Tables

**Table 1.** Sociodemographic Characteristics, Stratified by Total Monthly Income, of MSWs: Lima, Peru (March 2014).

Characteristics of the study participants	Total	Income Group 1 (US$0-US$137)	Income Group 2 (US$138-US$250)	Income Group 3 (US$251-US$429)	Income Group 4 (US$430-US$1,327)
	*N* = 209	*n* = 51	*n* = 57	*n* = 51	*n* = 50
*M* age (SD)	25.4 (6.2)	23.8 (20.0-30.0)	23.3 (20.4-27.5)	26.1 (21.3-28.9)	24.9 (22.0-28.8)
Completed high school*	122 (58%)	29 (57%)	30 (53%)	27 (53%)	36 (72%)
Self-described sexual role					
Insertive	139 (67%)	33 (65%)	41 (72%)	31 (61%)	34 (68%)
Receptive	9 (4%)	1 (2%)	2 (4%)	2 (4%)	4 (8%)
Both	61 (29%)	17 (33%)	14 (25%)	18 (35%)	12 (24%)
Self-described sexual orientation					
Heterosexual	50 (24%)	13 (25%)	15 (26%)	10 (20%)	12 (24%)
Homosexual	38 (18%)	12 (24%)	11 (19%)	8 (16%)	7 (14%)
Bisexual*	120 (58%)	26 (51%)	31 (54%)	32 (64%)	31 (62%)
Median years as MSW (IQR)	4.7 (2.1-9.4)	4.6 (2.3-10.4)	4.43 (1.8-9.7)	3.73 (1.8-9.1)	5.08 (2.1-9.0)
Alcohol abuse (CAGE)	130 (62%)	33 (65%)	36 (63%)	32 (63%)	29 (58%)
Drug use (past month)	79 (38%)	17 (33%)	24 (42%)	20 (39%)	18 (36%)
Stable living (pays monthly rent)	58 (28%)	13 (25%)	12 (21%)	13 (25%)	20 (40%)
Any condomless anal intercourse in past 3 months	89 (43%)	22 (43%)	22 (39%)	27 (53%)	29 (58%)
HIV positive*	51 (24%)	7 (14%)	14 (25%)	13 (25%)	17 (34%)
Recent syphilis, gonorrhea, or chlamydia infection	66 (32%)	8 (16%)	22 (39%)	16 (31%)	20 (40%)

Any STF^†^	103 (49%)	18 (35%)	28 (49%)	26 (51%)	31 (62%)

*Note*. Income grouping was based on total monthly income and was used for presentation, not statistical analysis purposes. Groups 1 to 4 cutoffs were chosen to create four similar-sized groups. All incomes are presented in US$ (exchange rate of 2.8 Peruvian Nuevo Soles = US$1). Income was treated as a continuous variable. Linear regression analysis was used for continuous characteristic variables (e.g., age) and student’s *t* test/ANOVA was used for categorical characteristic variables (e.g., high school completion). Any STI = HIV, syphilis, gonorrhea, or chlamydia infection. MSW = male sex workers; IQR = interquartile range; STI = sexually transmitted infections.

† p < .1

* p < .05

** p < .01

*** p < .001, for associations between study characteristics (first column) and total income.

**Table 2.** Breakdown of Monthly Income of Male Sex Workers: Lima, Peru (March 2014).

	Total	Income Group 1 (US$0-US$137)	Income Group 2 (US$138-US$250)	Income Group 3 (US$251-US$429)	Income Group 4 (US$430-US$1,327)
Characteristics of the study participants	*N* = 209	*n* = 51	*n* = 57	*n* = 51	*n* = 50
Median monthly income from SW (IQR)***	143 (71-250)	71 (32-107)	161 (89-179)	179 (89-286)	289 (179-464)
Median monthly income from non-SW (IQR)***	50 (0-186)	0 (0-7)	43 (0-107)	125 (0-214)	286 (143-357)
Median total monthly income (IQR)	250 (143-411)	86 (54-107)	196 (171-214)	321 (286-375)	536 (464-668)
Median number of sexual partners past 3 months (IQR)***	20 (10-42)	11 (8-26)	20 (7-40)	20 (10-50)	29 (14-60)
Median number of clients past 3 months (IQR)***	15 (8-37)	10 (5-20)	14 (7-32)	20 (10-40)	27 (12-56)
Median amount charged per encounter (IQR)**	11 (9-14)	11 (7-13)	11 (9-14)	11 (9-13)	11 (11-18)

*Note.* Income grouping was based on total monthly income and was used for presentation, not statistical analysis purposes. Groups 1 to 4 cutoffs were chosen to create four similar-sized groups. All incomes are presented in US$ (exchange rate of 2.8 Peruvian Nuevo Soles = US$1). Income was treated as a continuous variable. Linear regression analysis was used for continuous characteristic variables (e.g., age) and student’s *t* test/ANOVA was used for categorical characteristic variables (e.g., high school completion). SW = sex work; IQR = interquartile range.

† *p* < .1

* *p* < .05

** *p* < .01

*** *p* < .001, for associations between study characteristics (first column) and total income.

**Table 3.** Characteristics of the 72 Men Who Provided Spending Data: Lima, Peru (April 2015).

*M* age (*SD*)	27.7 (7.2)
Completed high school	47 (65%)
Self-described sexual role	
Insertive	41 (57%)
Receptive	6 (8%)
Both	25 (35%)
Self-described sexual orientation	
Heterosexual	12 (16%)
Homosexual	19 (26%)
Bisexual	41 (57%)
Median years as MSW (IQR)	6.1 (3.4-10.3)
Stable living (pays monthly rent)	28 (39%)
Any condomless anal intercourse in past 3 months	26 (36%)
HIV positive	24 (33%)
Recent syphilis, gonorrhea, or chlamydia infection	24 (33%)
Median monthly income from SW (IQR)	200 (100-400)
Median monthly income from non-SW (IQR)	123 (0-287)
Median total monthly income (IQR)	329 (184-433)

*Note.* MSW = male sex workers; IQR = interquartile range; SW = sex work.
